# A randomized controlled trial of nitrate supplementation in well-trained middle and older-aged adults

**DOI:** 10.1371/journal.pone.0235047

**Published:** 2020-06-23

**Authors:** Michael J. Berry, Gary D. Miller, Daniel B. Kim-Shapiro, Macie S. Fletcher, Caleb G. Jones, Zachary D. Gauthier, Summer L. Collins, Swati Basu, Timothy M. Heinrich

**Affiliations:** 1 Health and Exercise Department, Wake Forest University, Winston-Salem, North Carolina, United States of America; 2 Physics Department, Wake Forest University, Winston-Salem, North Carolina, United States of America; University of Alabama at Birmingham, UNITED STATES

## Abstract

**Purpose:**

Nitrate (NO_3_^-^), through its conversion to nitrite (NO_2_^-^) and nitric oxide, has been shown to increase exercise tolerance in healthy younger adults and older diseased patients. Nitrate’s effect in well-trained middle to older-aged adults has not been studied. Therefore, the purpose of this investigation was to examine the effects of a NO_3_^-^ rich beverage on submaximal constant work rate exercise time in well-trained middle to older-aged adults.

**Methods:**

This was a randomized controlled cross-over trial with 15 well-trained middle to older-aged adults, 41–64 year-old, who received one of two treatments (NO_3_^-^ rich beverage then placebo or placebo then NO_3_^-^ rich beverage), after which an exercise test at 75 percent of the subject’s maximal work rate was completed.

**Results:**

The NO_3_^-^ rich beverage increased plasma NO_3_^-^ and NO_2_^-^ levels by 260 μM and 0.47 μM, respectively (p<0.001). Exercise time was not significantly different (p = 0.31) between the NO_3_^-^ rich versus placebo conditions (1130±151 vs 1060±132 sec, respectively). Changes in exercise time between the two conditions ranged from a 55% improvement to a 40% decrease with the NO_3_^-^ rich beverage. Oxygen consumption and rating of perceived exertion were not significantly different between the two conditions.

**Conclusion:**

In middle to older-aged well-trained adults, NO_3_^-^ supplementation has non-significant, albeit highly variable, effects on exercise tolerance.

ClinicalTrials.gov Identifier: NCT03371966

## Introduction

Nitric oxide (NO) has been identified as an important biological messenger involved in a number of physiological processes including blood flow regulation, mitochondrial respiration, and cardiac and skeletal muscle contractility. It is produced endogenously from L-arginine and oxygen by NO-synthases (NOS) or the more recently identified reductive (non-NOS) nitrate (NO_3_^-^) → nitrite (NO_2_^-^) → NO pathway [1]. The production of NO via the NOS pathway is inhibited under hypoxic conditions, whereas the NO_3_^-^ → NO_2_^-^ → NO pathway is activated under hypoxic conditions. Once ingested, dietary NO_3_^-^ is reduced to bioactive NO_2_^-^ by commensal anaerobic bacteria found in the saliva and then further reduced to NO via multiple enzymatic and non-enzymatic pathways [2,3]. The NO_3_^-^ → NO_2_^-^ → NO pathway has been shown to affect a number of physiological processes that could lead to an improved exercise response following NO_3_^-^ ingestion [1]. However, the evidence supporting the benefits of NO_3_^-^ as an ergogenic aid is equivocal, and there are several factors that can influence its efficacy including, age, training level, dosage, and the mode, duration and intensity of the exercise [4].

Research with healthy younger recreationally active adults has repeatedly shown that ingestion of dietary NO_3_^-^ can reduce the oxygen cost of submaximal exercise and improve exercise performance during high intensity exercise [5,6]. Conversely, research with younger, well trained endurance athletes is equivocal, as some studies have failed to demonstrate an improvement in exercise performance following NO_3_^-^ ingestion [7–11] while others have demonstrated an ergogenic benefit [12–15]. There are several potential reasons that have been suggested for the conflicting results between trained and untrained individuals. First, it has been hypothesized that well-trained elite younger athletes may consume higher levels of dietary NO_3_^-^ as a result of the increased daily energy intake related to their increased daily energy expenditure [4]. Interestingly, Jonvik et al. have shown the median dietary intake of NO_3_^-^ in a large group of Dutch athletes to be 106 mg per day [16], a value not that different from what had been reported in the general Dutch population, 99 mg per day [17]. A second reason suggested is that these athletes have increases in NOS activity, thus obviating the need for the NO_3_^-^ → NO_2_^-^ → NO pathway [18]. Finally, elite endurance athletes exhibit a lower proportion of type II muscle fibers which have been hypothesized to be more responsive to NO_3_^-^ supplementation [19].

Nitric oxide metabolism has also been shown to be affected by age, and older adults have been shown to have impairments to the NOS pathway. More specifically, older adults have lower levels of L-arginine, the NOS substrate, and tetrahydrobiopterin, a NOS cofactor [20,21]. Additionally, aging is positively associated with increases in asymmetric dimethylarginine–an endogenous inhibitor of NOS [22]. We have shown that consumption of a supplement high in NO_3_^-^, such as beetroot juice, leads to elevated plasma NO_2_^-^ levels and may help restore NO metabolism in older adults; whereas a diet high in NO_3_^-^ without supplementation was insufficient at increasing plasma NO_2_^-^ levels [23]. As such, NO_3_^-^ supplementation has the potential to improve physical function and exercise tolerance in older adults. Our research, along with that of others, has shown NO_3_^-^ supplementation to have positive effects on physical function and exercise tolerance in older adults with chronic diseases [24–27]. Research examining the effects of dietary NO_3_^-^ on exercise performance in healthy older adults is limited. Kelly et al. demonstrated that three days of NO_3_^-^ rich beetroot juice consumption did not improve walking performance in a group of 12 healthy, active 60 to 70 year olds [28]. Additionally, Siervo et al. failed to find significant improvements in the physical function of healthy older adults following seven days of NO_3_^-^ supplementation [29]. Based on the fact that NO metabolism is affected by age, we hypothesized that NO_3_^-^ supplementation would increase exercise tolerance in middle to older-aged adults who engage in regular strenuous physical activity or competitive sports. Our primary objective of this investigation was to evaluate the effect of supplementation with a NO_3_^-^ rich beverage on exercise tolerance in healthy, active, well-trained middle to older-aged adults as compared to placebo supplementation.

## Methods

### Subjects

Twenty-nine individuals were screened for participation in the study (see below), and 15 of them between the ages of 41 and 64 completed the study. All subjects signed an informed consent approved by the Wake Forest University Institutional Review Board—approval number IRB00022914. Descriptive characteristics of the subjects completing the study are presented in Table 1. Inclusion criteria included the following: competitive runner or cyclist (must have competed in a running or cycling event within the previous year), between the ages of 40 and 65, able to pedal a stationary bike, engage in moderate physical activity for at least 150 minutes per week or engage in strenuous physical activity for at least 75 minutes per week, able to provide own transportation to study testing visits, able to consume study beverages, able to speak and read English and a willingness to provide informed consent and participate in the intervention. Individuals were excluded from the study if they were a current tobacco user, were participating in another research intervention, had contraindications for engaging in vigorous exercise, had a history of hypotension, diabetes, kidney stones, atrophic gastritis, thyroid disorder other than hypothyroidism, cardiovascular disease, chronic obstructive pulmonary disease, impaired liver or kidney function, inflammatory bowel disease or were taking any of the following medications: phosphodiesterase type 5 inhibitors, nitroglycerin or nitrate preparations, or proton pump inhibitors. The 15 subjects that completed the study all reported that they cycled regularly. Fourteen of the 15 had competed in a cycling event the previous year, and three had competed in running events.

**Table 1:** Descriptive characteristics of subjects completing the study.

	Females	Males	All
**n**	4	11	15
**Age (years)**	52 ± 9	48 ± 4	49 ± 6
**Body Mass (kg)**	54.6 ± 3.4	79.8 ± 11.0	73.1 ± 14.9
**Height (m)**	1.62 ± 0.03	1.77 ± 0.07	1.73 ± 0.09
**BMI (kg/m**^**2**^**)**	20.7 ± 1.8	25.3 ± 2.7	24.1 ± 3.2
**V̇O**_**2 peak**_ **(ml/kg/min)**	51.1 ± 5.0	51.9 ± 5.2	51.7 ± 5.0
**Work Rate Max (watts)**	220 ± 16	366 ± 41	327 ± 76

### Design and protocol

**Fig 1:**
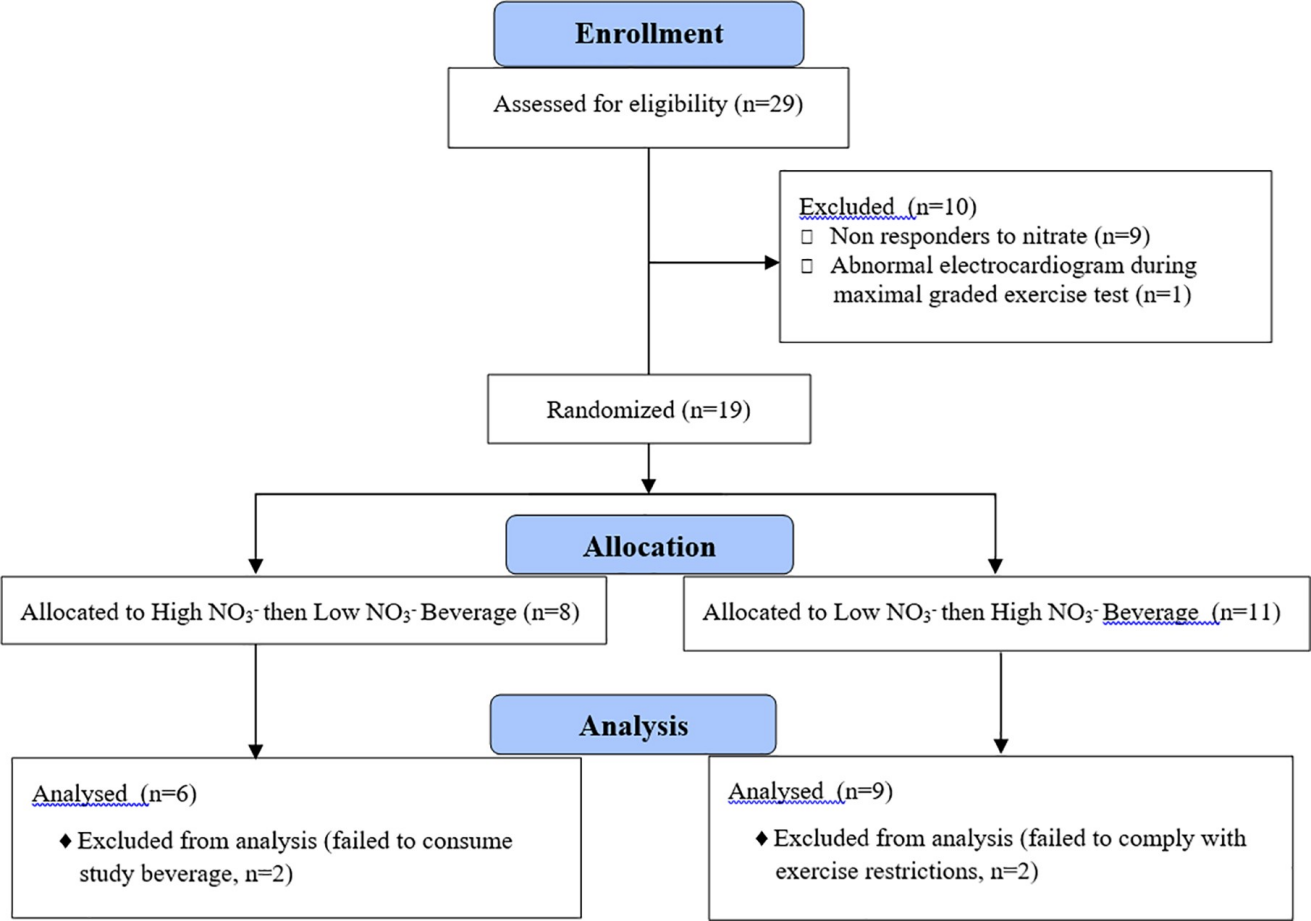
Subject flow through study.

This study was a double blind, placebo-controlled, randomized, cross over study with exercise tolerance as measured by submaximal constant work rate exercise time as the primary outcome. Data were collected at a private university in Winston-Salem, NC. Participants were recruited from the local community through advertisements placed on local running and cycling websites and Facebook pages. Participant recruitment and follow-up was from 12/17 through 12/19. This trial is registered with ClinicalTrials.gov under the title “Nitrate and Exercise Performance in Middle to Older Aged Adults” number NCT03371966. The flow of subjects through the various phases of the study is shown in the CONSORT flowchart in Fig 1.

**Fig 2:**
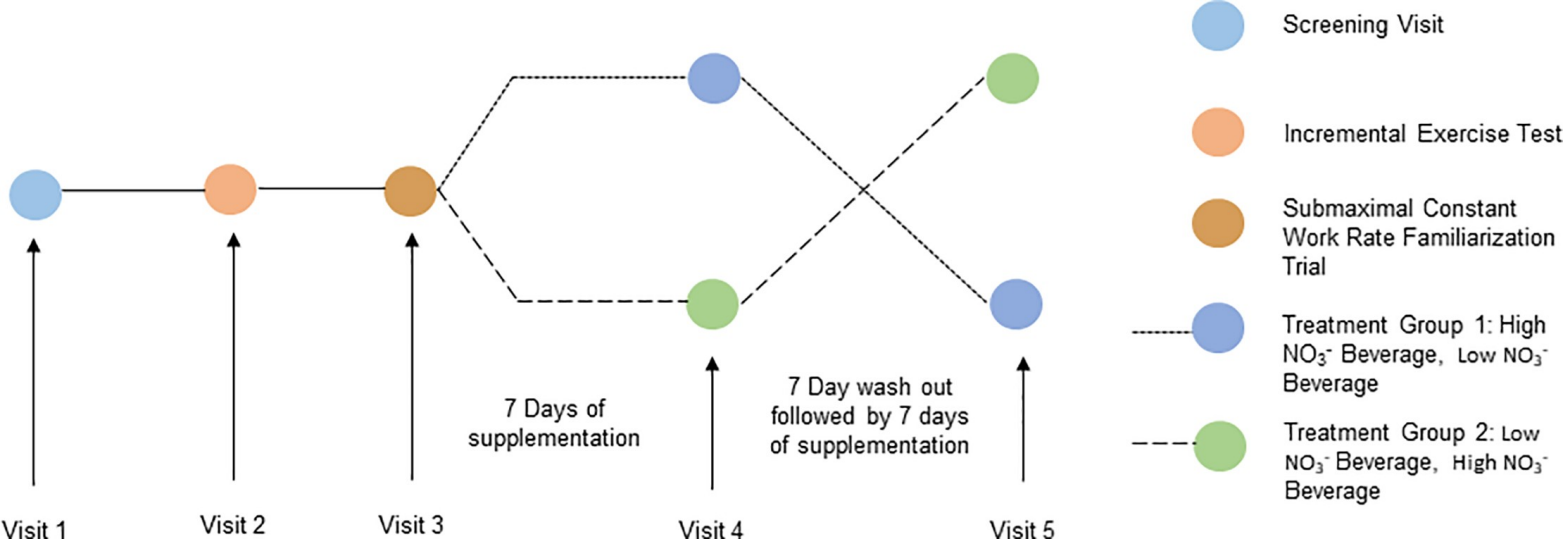
Schematic of study design.

Subjects completed one screening visit and four subsequent follow-up visits. A schematic of the study visits is show in Fig 2. During the screening visit (V1), a 3–4 ml venous blood sample was obtained to determine baseline plasma NO_3_^-^ and NO_2_^-^ levels. Subjects then consumed two servings (4 ounces) of a NO_3_^-^ rich dietary supplement (AMPED NOx; Isagenix International, LLC, Gilbert, AZ, USA) containing 9.9 mmoles of NO_3_^-^. Approximately two hours later, a second 3–4 ml venous sample was obtained for the determination of plasma NO_3_^-^ and NO_2_^-^ levels. Subjects that responded to the NO_3_^-^ dosing by exhibiting a 100 percent or greater increase in plasma NO_2_^-^ were asked to return for a second visit (V2). Previous research has shown a high degree of variability in NO_2_^-^ levels following NO_3_^-^ consumption [30]. In fact, James et al. raised the question as to whether studies examining exercise performance should aim for a certain percentage increase in nitrite levels [30]. Additionally, our previous research has shown that approximately 20 percent of older adults consuming NO_3_^-^ do not exhibit an increase in plasma NO_2_^-^ levels; therefore, we screened subjects to ensure that they responded to the NO_3_^-^ ingestion by exhibiting an increase in NO_2_^-^ levels [24,31]. Subjects were not aware of the exact purpose of the study, but were informed that we wished to examine the effect of two different beverages on exercise tolerance.

During V2, subjects performed an incremental exercise test on an electronically braked cycle ergometer (Lode Corival 2015, Netherlands) for the determination of their maximal work rate and peak oxygen consumption (V̇O_2 peak_). The test began with a three-minute warm up at either 40 or 50 watts (female and male, respectively) after which time the work rate was increased by 20 or 30 watts (female and male, respectively) each minute until volitional exhaustion. The subject’s maximal work rate and V̇O_2 peak_ were determined based on the highest work rate completed for a full minute. Oxygen consumption (V̇O_2_) was measured continuously throughout the test. At the conclusion of V2, subjects were scheduled for visit 3 (V3) and provided with a list of foods high in NO_3_^-^ that they were to avoid for 48 hours prior to all subsequent visits. Subjects were also instructed to avoid using antibacterial mouthwash for the duration of the study, to maintain similar exercise routines and diets prior to all subsequent visits and to avoid strenuous exercise for 48 hours prior to all subsequent visits. Additionally, subjects were instructed to arrive at the lab at least 2 hours postprandial and to have not consumed alcohol for 48 hours prior to the visit.

Visit 3 consisted of a familiarization submaximal constant work rate exercise test on the cycle ergometer at 75 percent of the maximal work rate achieved during the incremental exercise test at V2. The subjects completed a five-minute warm-up at 50 percent of their maximal work rate, after which time the work rate was increased to 75 percent of their maximal work rate. The subjects were asked to maintain that work rate for as long as they could. If the subjects were able to maintain the work rate for 30 minutes, the resistance was increased by five percent of their maximal work rate every 15 minutes thereafter until the subjects were no longer able to maintain a cadence ≥60 rpm at the prescribed work rate. During this test, V̇O_2_ and the tissue saturation index were measured continuously. Every five minutes throughout the test and at the end of the test, measures of blood pressure, heart rate and ratings of perceived exertion were obtained. Following completion of the exercise test, the subjects were randomized to one of the two treatment arms: seven days of a high NO_3_^-^ beverage, a seven-day washout period and then seven days of a low NO_3_^-^ beverage (placebo); or seven days of a placebo beverage, a seven-day washout period and then seven days of a high NO_3_^-^ beverage. Randomization was done using a computerized program to assign individuals, in the order that they arrived, to the treatments arms using simple random assignment. Subjects were given a sealed box containing the seven-day supply of one of the two beverages. Investigators collecting study related data and supplying the beverages to the subjects were unaware of the beverage being supplied to the subjects. For the seven days leading up to visit 4 (V4) and visit 5 (V5), subjects received a text message reminding them to consume their study beverage.

Visit 4 consisted of a submaximal constant work rate exercise test at 75 percent of the maximal work rate and followed the same procedures described for V3. Subjects received a text message the morning of their V4 reminding them to consume their beverage two hours prior to the scheduled visit. Upon arrival for V4, a venous blood sample was obtained for the determination of plasma NO_3_^-^ and NO_2_^-^. Once the blood sample had been obtained, the exercise test was performed. At the completion of the exercise test, V5 was scheduled, and subjects were provided with their respective beverages that were given a sealed box containing a seven-day supply of one of the two beverages. Visit 5 followed the same procedures as described for V4.

The high NO_3_^-^ beverage provided to the subjects was AMPED NOx, (Isagenix International LLC, Gilbert, Arizona, USA). Subjects were provided with 14 two-ounce bottles, and, during the high NO_3_^-^ beverage supplementation period, the subjects were instructed to consume two bottles each day. Four ounces of the AMPED NOx contained 9.9 mmoles of NO_3_^-^. The low NO_3_^-^ beverage provided to the subjects was V8 Purple Power, Campbells Food Service (Camden, New Jersey, USA). Subjects were provided with three 12-ounce bottles and a four-ounce measuring cup. During the low NO_3_^-^ beverage supplementation period, the subjects were instructed to consume four ounces each day. For both the low and the high nitrate beverage trials, the subjects were instructed to consume the four-ounce dose at the same time of day throughout the seven-day supplementation period. Four ounces of the V8 Purple Power contained 0.5 mmoles of NO_3_^-^. The V8 Purple Power was selected as the placebo because it had minimal levels of NO_3_^-^ and had a similar consistency and color. All bottles supplied to the subjects had the labels removed and subjects were instructed not to share any information about their beverage with the study staff. Four ounces of the AMPED NOx contained 60 calories, 12 grams of total carbohydrates, 2 grams of fiber and 4 grams of sugar. Four ounces of the V8 Purple Power contained 26.7 calories, 6.3 grams of total carbohydrates, 0 grams of fiber and 5.3 grams of sugar. Both beverages were assayed for their NO_3_^-^ levels using the ENO 20 NOx analyzer, Eicom.

### Study outcomes

The primary outcome for this study was exercise time, in seconds, during the submaximal constant work rate tests at V4 and V5. Exercise time was recorded from the start of the 75 percent of maximal work rate until the subject could no longer maintain a cadence of 60 rpm or above or volitional exhaustion. Standardized encouragement was provided throughout all tests.

Secondary outcomes included plasma NO_3_^-^ and NO_2_^-^ levels, V̇O_2_, tissue saturation index, heart rate, rating of perceived exertion, and systolic and diastolic blood pressures. The evaluation of the secondary outcomes V̇O_2_, tissue saturation index, heart rate, rating of perceived exertion, and systolic and diastolic blood pressures was done at three time points during the constant work rate tests at V4 and V5. These were at five minutes after the start of exercise, iso-time exercise and at the end of exercise. Iso-time exercise was defined as the last minute of the shortest exercise time during either V4 or V5. Values from that time were then matched with those obtained at the same time from the longer duration exercise test. End of exercise blood pressures were measured as the last set of data collected during each of the tests. Oxygen consumption and tissue saturation index were the final complete 60-second values collected. Heart rate and rating of perceived exertion were obtained at the end of exercise.

All blood samples were obtained from the subject’s antecubital vein and collected in a 4 mL lithium heparin vials for the determination of plasma NO_3_^-^ and NO_2_^-^ levels. The blood samples were centrifuged at 4000 rpm (2006 g) for three minutes, the plasma was removed and stored in a -80º C freezer for subsequent analysis. Nitrate and NO_2_^-^ levels were determined using a NOx analyzer ENO-20 (EICOM, Kyoto, Japan), designed specifically for measuring NO_3_^-^ and NO_2_^-^ in fluid samples and based on the colorimetric Greiss assay. Standard curves were obtained for all measurements and used for quantitative measurements. Oxygen consumption data was collected using a COSMED K5 portable cardiorespiratory gas exchange system (COSMED, Rome, Italy). Continuous wave near infrared spectroscopy (NIRS) was used to measure the tissue saturation index. The NIRS probe (PortaMon, Artinis Medical Systems, Elst, Netherlands) was placed over the belly of Vastus Lateralis of the right leg. Probe position was measured and recorded to ensure identical placement on subsequent visits. The probe was attached to the skin using adhesive tape and then wrapped with a black elastic band to prevent movement and negate the influence of ambient light. The tissue saturation index was calculated using the manufacturer’s Oxysoft software. Data were sampled at 10 Hz and then averaged every second. The tissue saturation index is reported as differences between the last 30 seconds of the resting value and the last 30 seconds of the fifth minute of the 75% work rate. Perceived exertion was measured with the Borg 10 point scale. Blood pressure was measured manually using the auscultatory method. Only a single measurement was made at each time point throughout the exercise tests. In most instances, blood pressure was measured in the left arm of the subject unless blood had been drawn from that arm for the NO_3_^-^ and NO_2_^-^ determinations. Heart rate was monitored using a Garmin HRM-Dual heart rate monitor (Garmin, Kansas City, USA).

### Statistical analyses

Data were analyzed using SPSS version 25.0. Normality of data were first assessed by visual inspection of normal quantile plots. Suspected deviations from normality were subsequently tested using a Shapiro Wilk test. Due to non-normality of data, the Friedman test was used to test for differences in NO_3_^-^ and NO_2_^-^ values at the various sampling time points. Dependent t-tests were used to test for differences in outcomes between the high and low NO_3_^-^ beverage trials. Due to non-normality of end exercise systolic blood pressure and end exercise RPE values, a Wilcoxon test was used to test for differences in outcomes between the high and low NO_3_^-^ beverage trials. Associations between changes in exercise time for the high and low NO_3_^-^ beverage trials and changes in NO_3_^-^ and NO_2_^-^ levels for the high and low NO_3_^-^ beverage trials and changes in V̇O_2_ for the high and low NO_3_^-^ beverage trials were examined using the Pearson correlation coefficient. The relationship between V̇O_2 peak_ and changes in NO_3_^-^ and NO_2_^-^ from pre to post beverage consumption at VI were also assessed using the Pearson correlation coefficient. All tests were two-sided and significance was set at p < 0.05. Outcome variables are reported as means with 95% confidence intervals (CI) or medians with 5^th^, 25^th^, 75^th^ and 95^th^ percentiles. Sample size was based on our previous experience testing older adults using constant work rate tests [24]. More specifically, because we were able to achieve statistical significance (p = 0.031) with an effect size of 0.5 using 15 older adults in that previous study, that was the sample size we targeted.

## Results

**Fig 3:**
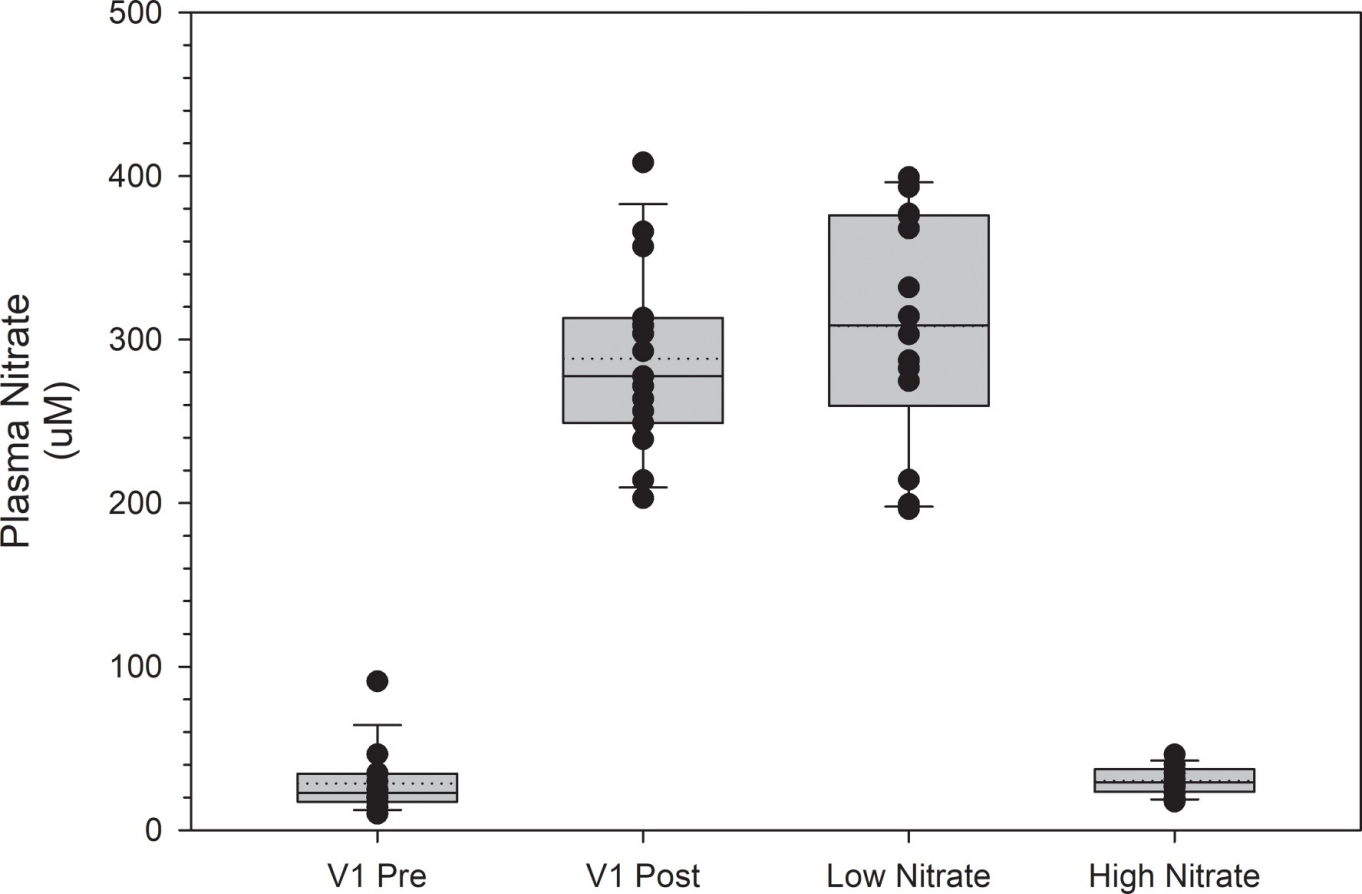
Plasma NO3- levels at visit 1 (V1) and the high and low NO3- beverage trials. Post NO_3_^-^ levels at V1 were obtained two hours post consumption of a high NO_3_^-^ beverage. Plasma NO_3_^-^ levels at the high and low NO_3_^-^ beverage trials were obtained two hours post consumption of the respective beverage. Plasma NO_3_^-^ levels were not significantly different when comparing the high NO_3_^-^ beverage trial values to the post consumption values at V1 (p = 1.0) or when comparing the low NO_3_^-^ beverage trial values to the pre-consumption values at V1 (p = 1.0). Values are represented as median values with box ends representing the 25^th^ and 75^th^ percentiles and error bars representing the 5^th^ and 95^th^ percentiles. Dotted lines represent the mean values, and individual dots represent subject values.

**Fig 4:**
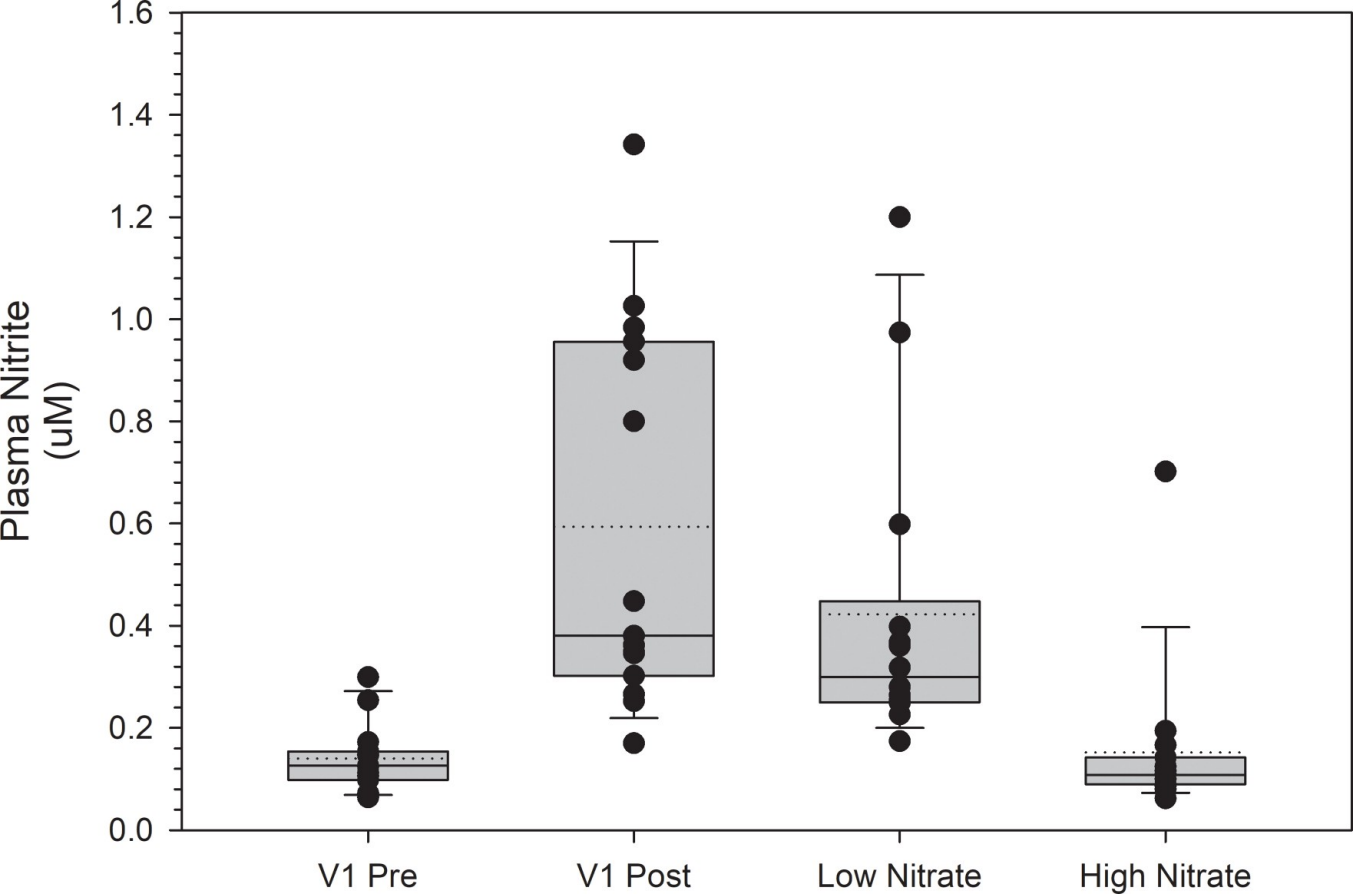
Plasma NO2- levels at visit 1 (V1) and the high and low NO3- beverage trials. Post NO_2_^-^ levels at V1 were obtained two hours post consumption of a high NO_3_^-^ beverage. Plasma NO_2_^-^ levels at the high and low NO_3_^-^ beverage trials were obtained two hours post consumption of the respective beverage. Plasma NO_2_^-^ levels were not significantly different when comparing the high NO_3_^-^ beverage trial values to the post consumption values at V1 (p = 1.0) or when comparing the low NO_3_^-^ beverage trial values to the pre-consumption values at V1 (p = 1.0). Values are represented as median values with box ends representing the 25^th^ and 75^th^ percentiles and error bars representing the 5^th^ and 95^th^ percentiles. Dotted lines represent the mean values, and individual dots represent subject values.

Plasma NO_3_^-^ and NO_2_^-^ levels pre and post consumption at V1 and at the high and low NO_3_^-^ beverage trials are shown in Figs 3 and 4, respectively. Following consumption of the high NO_3_^-^ beverage at V1, plasma NO_3_^-^ levels increased by 260 μM (p < 0.001). Plasma NO_3_^-^ levels at the high NO_3_^-^ beverage trial were not significantly different from post consumption plasma NO_3_^-^ values at V1 (p = 1.0). Plasma NO_3_^-^ levels at the low NO_3_^-^ beverage trial were not significantly different from pre-consumption NO_3_^-^ values at V1 (p = 1.0). Following consumption of the high NO_3_^-^ beverage at V1, plasma NO_2_^-^ levels increased by 0.47 μM (p < 0.001). Plasma NO_2_^-^ levels at the high NO_3_^-^ beverage trial were not significantly different from post consumption plasma NO_2_^-^ values at V1 (p = 1.0). Plasma NO_2_^-^ levels at the low NO_3_^-^ beverage trial were not significantly different from pre-consumption plasma NO_2_^-^ values at V1 (p = 1.0). The relationship between V̇O_2 peak_ and changes in NO_3_^-^ and NO_2_^-^ levels from pre to post beverage consumption at V1 were non-significant (r = -0.047 and 0.095, and p = 0.868 and 0.736, respectively).

**Fig 5:**
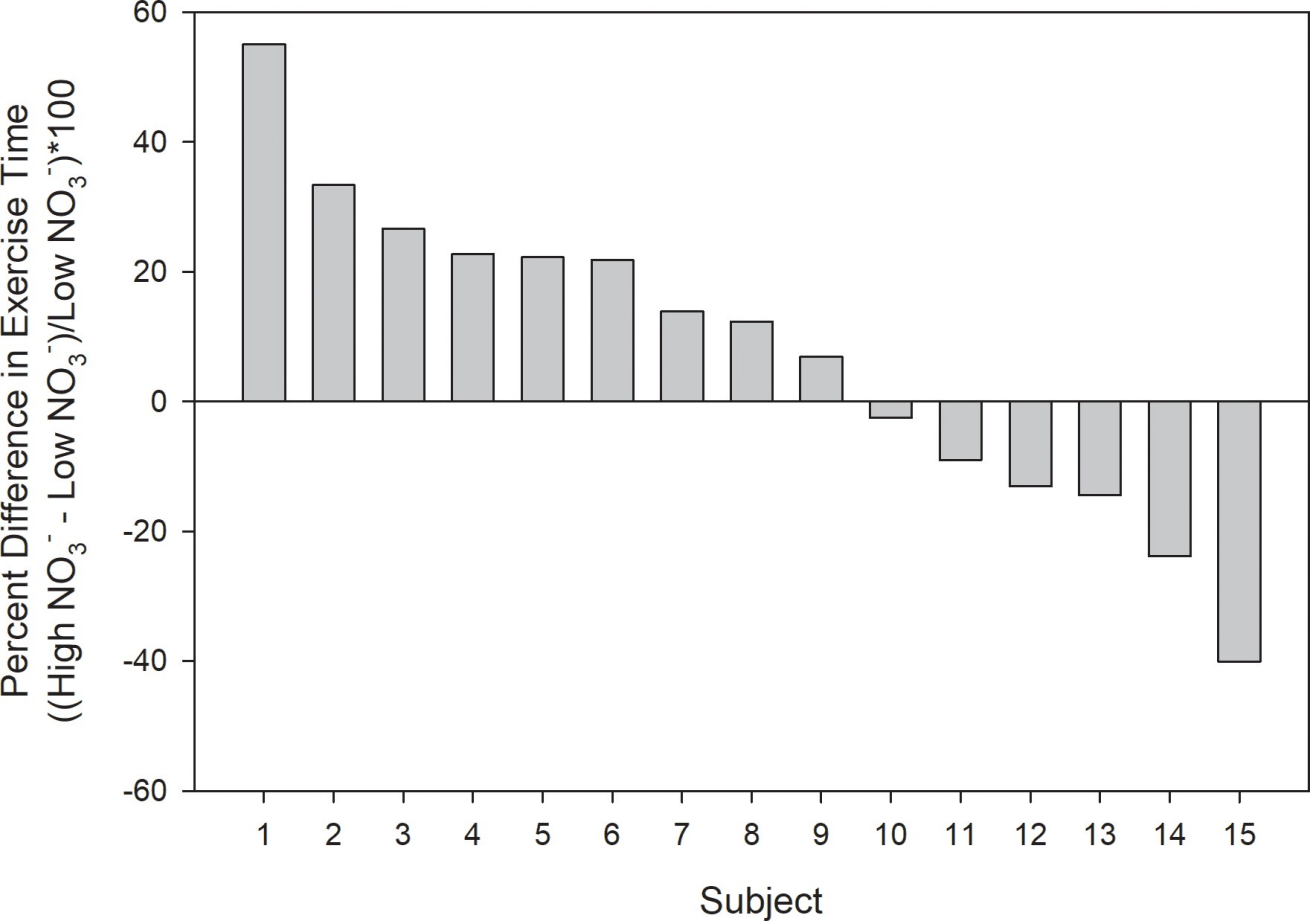
Individual subject responses. Bars represent individual subject responses that ranged from a 55 percent improvement in exercise time during the high NO_3_^-^ beverage trial as compared to the low NO_3_^-^ beverage trial to as low as a 40 percent decrement in performance.

Exercise time between the high NO_3_^-^ versus low NO_3_^-^ beverage trials (1131 [806–1456] versus 1060 [778–1342] seconds, respectively) was not significantly different (p = 0.31). Individual differences in exercise time between the high NO_3_^-^ and low NO_3_^-^ beverage trials, expressed as a percent of the low NO_3_^-^ beverage trial time, are shown in Fig 5. Changes in exercise time between the two trials ranged from as great as a 55% improvement with the high NO_3_^-^ beverage to a 40% decrease in time. Interestingly, the two subjects that had the largest decreases in exercise time with the high NO_3_^-^ beverage (24 and 40%) were both diagnosed with hypothyroidism and were each taking 0.75 mg of levothyroxine per day. The correlations between differences in exercise time for the high and low NO_3_^-^ beverage trials and differences in NO_3_^-^ and NO_2_^-^ levels for the high and low NO_3_^-^ beverage trials were not significant (r = 0.157 and -0.173, and p = 0.593 and 0.554, respectively). Additionally, the correlations between changes in exercise time for the high and low NO_3_^-^ beverage trials and changes in V̇O_2_ for the high and low NO_3_^-^ beverage trials at five minutes and iso-time exercise were not significant (r = -0.050 and 0.105, and p = 0.859 and 0.708, respectively).

Mean values for systolic and diastolic blood pressure, oxygen consumption, heart rate and rating of perceived exertion at five minutes into exercise, at iso-time exercise and end exercise are shown in Table 2. Diastolic blood pressure at five minutes was 4.2 mm Hg lower during the low NO_3_^-^ beverage trial as compared to the high NO_3_^-^ beverage trial (P = 0.049). Heart rate at iso-time was 2 beats per minute lower during the high NO_3_^-^ beverage trial as compared to the low NO_3_^-^ beverage trial (P = 0.007). None of the other exercise variables were significantly different between the high and low NO_3_^-^ beverage trials. The tissue saturation indexes for the high and low NO_3_^-^ beverage trials were not significantly different from one another (-7.8 [-10.6 - -5.0] vs. -7.8 [-11.0 - -4.6] %, respectively; p = 0.990).

**Table 2:** Exercise responses during the high and low NO3- beverage trials.

	High NO_3_^-^ Beverage Trial	Low NO_3_^-^ Beverage Trial	P
**Systolic Blood Pressure, mmHg**			
**5 Minute**	185 [174–196]	185 [176–195]	0.988
**Iso-Time**	191 [180–203]	190 [179–201]	0.835
**End Exercise**	196 [182–210]	193 [183–204]	0.851
**Diastolic Blood Pressure, mmHg**			
**5 Minute**	79 [76–82]	75 [71–79]	0.049
**Iso-Time**	77 [74–81]	74 [69–79]	0.253
**End Exercise**	78 [74–82]	75 [71–80]	0.443
**Oxygen Consumption ml**^.^**kg**^**-1.**^**min**^**-1**^			
**5 Minute**	43.9 [40.8–47.1]	43.1 [40.0–46.3]	0.499
**Iso-Time**	47.7 [44.4–50.9]	46.4 [42.5–50.3]	0.549
**End Exercise**	47.5 [44.5–50.5]	46.8 [43.0–50.7]	0.303
**Heart Rate (bpm)**			
**5 Minute**	155 [147–162]	153 [143–163]	0.566
**Iso-Time**	166 [159–172]	168 [161–174]	0.007
**End Exercise**	168 [162–174]	169 [162–175]	0.605
**Rating of Perceived Exertion (1–10)**			
**5 Minute**	5.1 [4.2–6.1]	5.6 [4.6–6.5]	0.097
**Iso-Time**	8.8 [8.2–9.4]	8.7 [8.0–9.4]	0.796
**End Exercise**	9.1 [8.5–9.7]	9.3 [8.8–9.8]	0.168

Values are means and 95% CI at five minutes into exercise, iso-tme exercise and end exercise at 75 percent of maximal capacity are shown for each of the trials.

None of the subjects experienced any harms or detrimental effects as a result of participating in the trial.

## Discussion

The results of this study showed that well-trained middle to older aged adults supplementing with a NO_3_^-^ rich beverage for a seven-day period did not experience a statistically significant improvement in exercise tolerance when compared to supplementing with a low NO_3_^-^ beverage. However, examination of individual subject responses showed a highly variable response to constant work rate exercise with some subjects showing large improvements in performance with high NO_3_^-^ supplementation and others showing a reduced exercise tolerance, in particular those with hypothyroidism. Differences in exercise blood pressure and oxygen consumption between the high and low NO_3_^-^ trials measured at different time points were minimal. The only significant differences were diastolic blood pressure at five minutes was higher and heart rate at iso-time was lower during the high NO_3_^-^ trial.

### Constant workrate exercise time

In this cohort of highly fit middle to older aged adults, we did not find a statistically significant increase in constant workrate exercise time. However, the 79 second increase in exercise time with the high NO_3_^-^ beverage may be a meaningful improvement for this population. Future research using a time trial protocol or multiple constant work rate tests is needed to establish the practical significance of this improvement. Previous studies with younger, recreationally active subjects using constant work rate tests have reported the improvements in exercise time during constant work rate tests to range between 12 and 25 percent [5,6,32]. Studies of older adults with various comorbidities have also reported similar findings. Studies with COPD patients, peripheral artery disease patients and those with heart failure have reported 8, 18 and 24 percent improvements in exercise time, respectively, with NO_3_^-^ supplementation [24–26]. It should be noted, however, that these are not consistent findings. Other studies with older, clinical populations have failed to find improvements in exercise performance with NO_3_^-^ supplementation [33,34].

As previously mentioned, the variation in the degree of improvement in response to the NO_3_^-^ supplementation seen among the subjects in this study was high. Previous studies with well-trained subjects have reported similar findings, and it has been suggested that with respect to exercise performance there are responders and non-responders to NO_3_^-^ supplementation [8,10,11]. While it is generally believed that NO_3_^-^ supplementation will improve exercise performance in younger recreationally active individuals, the results are equivocal when examining NO_3_^-^ supplementation and exercise performance in athletes [4]. There are studies showing improvements in performance with NO_3_^-^ supplementation in athletes [12–15], whereas others have failed to find improvements [7–11]. Interestingly, while Boorsma [8] and Christensen [10] both failed to find improvements in overall group mean performance, both studies reported that 20 to 25 percent of their subjects were positive responders to NO_3_^-^ supplementation when examining exercise performance. Presently, it is unclear as to the reason why some well-trained individuals will respond to NO_3_^-^ supplementation, whereas others will not; however, a number of reasons have been proposed. Wilkerson et al. suggested that highly trained individuals have increased NOS activity which might attenuate the influence of the NO_3_^-^ → NO_2_^-^ → NO pathway [11]. They reported baseline NO_2_^-^ levels close to 0.4 μM—more than double what we measured in our subjects and higher than what is typically reported. This difference may be due to the older age of our subjects and the associated reductions in nitrate metabolism that occur with aging. Porcelli and colleagues found that after NO_3_^-^ supplementation there were inverse relationships between the subject’s V̇O_2 max_ and reduced oxygen cost of submaximal exercise and improvement in three kilometer time trial performance [35]. They also found that subjects with a higher V̇O_2 max_ had attenuated increases in plasma NO_2_^-^ and NO_3_^-^ following NO_3_^-^ supplementation. We failed to find a significant relationship between V̇O_2 max_ and changes in either plasma NO_2_^-^ or NO_3_^-^ following NO_3_^-^ supplementation, which again may have been due to the age or the homogeneous fitness level of our subjects. It has also been suggested that physiological adaptations to long-term endurance training may diminish the efficacy of NO_3_^-^ supplementation [11]. As previously mentioned, all our subjects were well-trained individuals with high aerobic fitness levels. Because endurance training increases capillary density in skeletal muscle, this may preserve muscle oxygenation and consequently diminish the likelihood of developing a hypoxic environment in the muscle. The reduction of NO_2_^-^ to NO is enhanced in hypoxic environments, whereas the production of NO from the NOS pathway requires oxygen [1]. Therefore, the increased availability of oxygen may limit the reduction of NO_2_^-^ to NO and favor the NOS pathway. Nitrate supplementation has also been shown to increase muscle force production [36]. As such, differences in the proportion of type I and II fibers in higher fit individuals could also account for some of the variation. Finally, polymorphisms in the NOS gene could also account for some of the variation in exercise performance and/or the response to NO_3_^-^ supplementation. Montesanto et al. noted an association between inducible NOS genotype and physical performance suggesting that genetic variability of NOS may affect exercise performance [37].

### Effects of dietary nitrate supplementation on plasma nitrite levels

A unique aspect of this study was that we screened subjects for their ability to convert NO_3_^-^ to NO_2_^-^ prior to randomizing them into the study. There is significant variation in the plasma levels of NO_3_^-^ and NO_2_^-^ following consumption of dietary NO_3_^-^ [30]. We, along with others, have shown that nearly 20 percent or more of adults do not exhibit a significant increase in plasma NO_2_^-^ levels following consumption of a high NO_3_^-^ beverage [11,24,31]. Additionally, Wilkerson et al. reported that the exercise performance was only improved in those subjects who had increases in NO_2_^-^ levels following supplementation [11]. There are several possible reasons for the high variability in the conversion of dietary NO_3_^-^ to NO_2_^-^. First, differences in the abundance of species of the oral bacteria responsible for the conversion of NO_3_^-^ to NO_2_^-^ has been suggested to account for some of this variability [30]. Additionally, Porcelli and colleagues have shown an inverse relationship between aerobic fitness levels and increases in NO_3_^-^ and NO_2_^-^ following NO_3_^−^ supplementation [35]. We did not find such a relationship in this study, which may have been due to the lack of variability in the aerobic fitness levels of our subjects. Our subjects were all well-trained individuals, and when comparing their fitness levels to age and sex-matched norms, eight were at or above the 95^th^ percentile, three were at or above the 90^th^ percentile and four were at or above the 75^th^ percentile [38]. In this study, subjects ingested 9.9 mmol of NO_3_^-^ daily for seven days. Despite seven days of NO_3_^-^ consumption, NO_2_^-^ levels were not significantly different between the V1 post NO_3_^-^ consumption values and those obtained on day seven of the high NO_3_^-^ supplementation trial. While it might seem that a single dose of NO_3_^-^ may be as beneficial as seven days of supplementation, previous trials showing improved economy of oxygen utilization with NO_3_^-^ have used supplementation periods of at least three days [39,40]. It is unclear as to whether a single dosing would improve oxygen utilization efficiency. Previous studies have shown this level of consumption sufficient to increase NO_2_^-^ levels and improve exercise performance in younger highly trained athletes [13] and younger recreationally active men [5]. Following consumption of the high NO_3_^-^ beverage, plasma NO_3_^-^ and NO_2_^-^ levels were increased by 934 and 428 percent, respectively, similar to previously reported finding in both younger [15] and older adults [24].

### Exercise economy

Despite increases in NO_2_^-^, we failed to note differences in submaximal V̇O_2_, and we did not find a relationship between changes in submaximal V̇O_2_ and changes in exercise duration when comparing the high and low NO_3_^-^ beverage trials. These results would suggest that an improvement in exercise economy was not the mechanism responsible for the increase in exercise duration noted in some of our subjects. Rokkendal-Lausch et al. reported similar findings in their young well-trained cyclists [15]. They found that while NO_3_^-^ supplementation did improve time trial performance, it had no effect on exercise economy as measured by the power output to V̇O_2_ ratio. A number of other studies with well-trained athletes and healthy older adults have reported similar findings [8,10,29]. In contrast to our findings, Lansley et al. [14] and Wilkerson et al. [11] both reported improved exercise economy during time trial test with younger well-trained subjects following NO_3_^-^ supplementation. Additionally, a reduced O_2_ cost of exercise following NO_3_^-^ supplementation has been reported with young healthy recreationally active subjects [6].

### Blood pressure

In addition to a lack of differences in submaximal exercise V̇O_2_, we did not find differences in blood pressure or blood flow between the two trials. The exception to this was the diastolic blood pressure at minute 5 which was higher during the high NO_3_^-^ beverage trial. It is unclear as to why this may have occurred. The overall lack of significant blood pressure differences is supported by Oggioi et al. who failed to find differences in either systolic or diastolic blood pressure of healthy older adults at rest or during an incremental exercise test when comparing a NO_3_^-^ rich beverage to a placebo [41]. Siervo et al. also reported no differences in either the resting systolic or diastolic blood pressure of healthy older adults when comparing a NO_3_^-^ rich or a NO_3_^-^ depleted beverage [29]. In contrast, Kelly et al. found decreases in both resting systolic and diastolic blood pressure of healthy older adults when comparing a NO_3_^-^ rich to a NO_3_^-^ depleted beverage [28]. Significant reductions in systolic and diastolic blood pressure directly linked to NO_3_^-^ ingestion have been reported in young healthy adults [42] and older adults with comorbidities [24,26]. Reasons for the differences among the various studies are not clear. However, it may be due to one or more of the reasons cited above for the differences in exercise time reported among the various studies.

### Nitrate and hypothyroidism

It is unknown whether NO_3_^-^ supplementation negatively impacts hypothyroid patients’ exercise tolerance, or if the large decreases we noted in the two subjects with hypothyroidism was a fortuitous finding of this study. Previous research has shown that acute NO_3_^-^ supplementation does not affect thyroid levels in healthy individuals [43,44], and we are unaware of any data showing acute NO_3_^-^ supplementation to negatively affect exercise performance in hypothyroid patients. However, there is indirect evidence to suggest that NO_3_^-^ supplementation may negatively affect thyroid hormone levels and, therefore, exercise performance [45,46]. Epidemiological reports suggest a weak link between chronic increases in dietary NO_3_^-^ and increases in the prevalence of hypothyroidism and decreased thyroid hormone levels [45]. Furthermore, exercise intolerance has been noted in both treated and untreated hypothyroid patients [46]. Further studies are needed to more clearly define the role NO_3_^-^ plays in the exercise response of individuals with hypothyroidism.

### Strengths and limitations

The strengths of our study include the following: It was a double-blind, randomized crossover trial. We had subjects participate in a familiarization trial prior to the high and low NO_3_^-^ beverage trials. We recruited both men and women into the study, and we verified the NO_3_^-^ content of both study beverages using an analyzer designed specifically for measuring NO_3_^-^ and NO_2_^-^. Despite the many strengths of the study, it was not without its limitations. First, two different beverages were used, and they were packaged differently. While we measured the NO_3_^-^ content of both beverages, we did not measure levels of other ergogenic bioactive compounds such as betaine, polyphenols, quercetin or resveratrol. A second limitation of the study is we used an open-ended test rather than a time trial with our subjects. Open-ended test where subjects are required to exercise at a constant work rate for as long as possible have been criticized due to the greater variation in performance time versus closed-end tests where subjects are required to cover a given distance in the quickest time possible or ride for a given time period and told to complete the greatest distance possible [47]. However, it should be noted that not all studies using open-ended tests have reported large variations in exercise time [48], and studies have reported differences in exercise time when evaluating the effects of other nutritional supplements [49]. We chose the open-ended test as it has been shown to be responsive to NO_3_^-^ supplementation, and we wished to hold the work rate constant to determine if there was an increase in exercise economy.

## Conclusions and recommendations

In conclusion, we found that chronic supplementation with a NO_3_^-^ rich beverage did increase NO_3_^-^ and NO_2_^-^ levels in middle to older-aged adults; however, it did not appear to have a significant effect of exercise tolerance. Further research is needed to determine if dietary NO_3_^-^ will affect exercise performance in individuals with hypothyroidism or if this was a singular finding in this investigation. Additional research is also needed to determine what factors differentiate exercise responses between high and low responders relative to NO_3_^-^ supplementation. Finally, the effect of nitrate supplementation on time trial performance in a group of high-fit, well-trained older adults should be investigated.

## Supporting information

S1 Checklist CONSORT 2010 checklist of information to include when reporting a randomised trial*. (DOC)

S1 File (PDF)

S1 Data (XLSX)
