# Correction: A randomized controlled trial of nitrate supplementation in well-trained middle and older-aged adults

**DOI:** 10.1371/journal.pone.0238271

**Published:** 2020-08-20

**Authors:** Michael J. Berry, Gary D. Miller, Daniel B. Kim-Shapiro, Macie S. Fletcher, Caleb G. Jones, Zachary D. Gauthier, Summer L. Collins, Swati Basu, Timothy M. Heinrich

Fig 3 and Fig 4 are incorrect. On the x-axis, the tick label "Low Nitrate" should be "High Nitrate,” and the tick label "High Nitrate" should be "Low Nitrate." The authors have provided corrected versions here.

**Fig 3:**
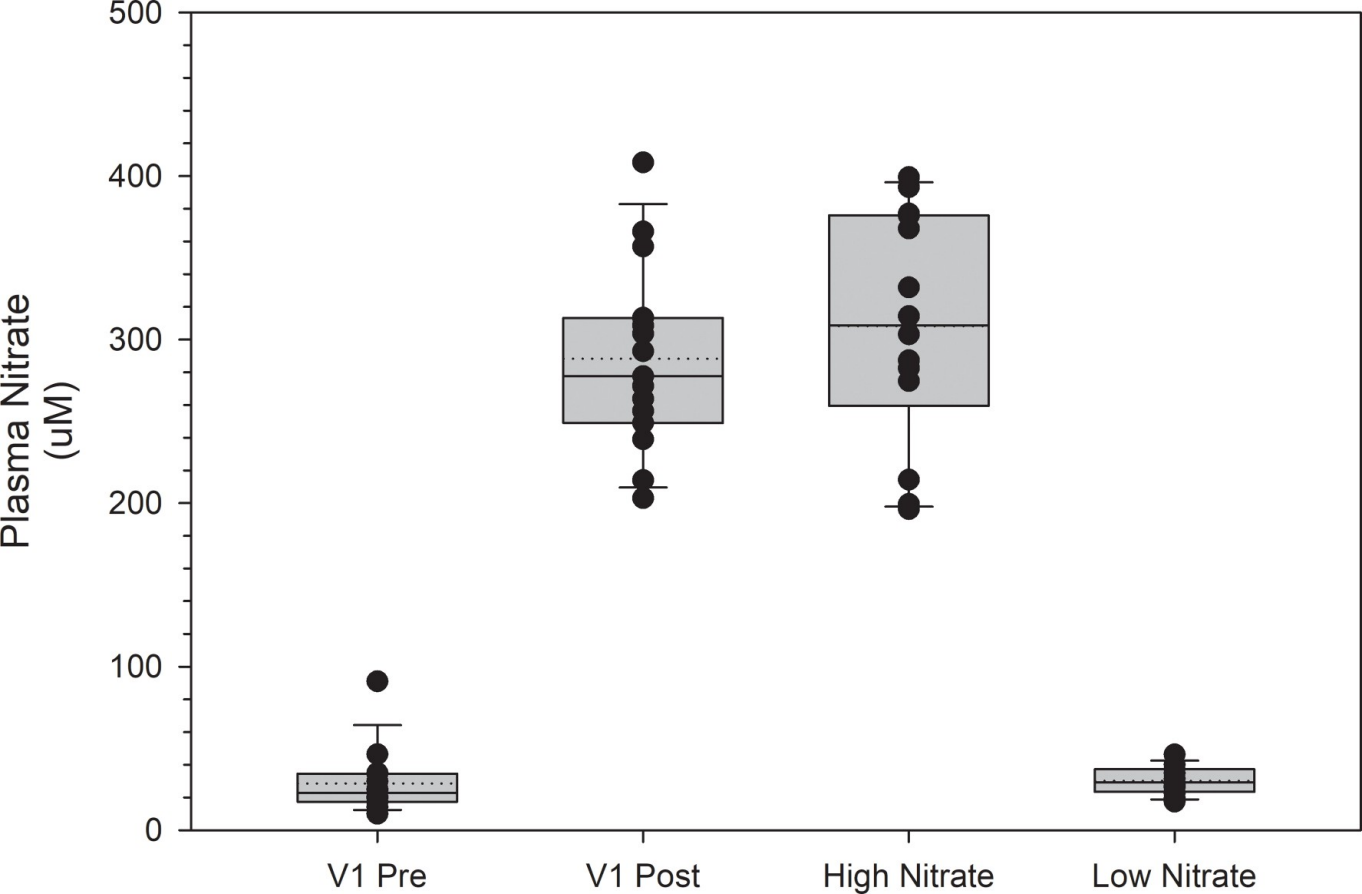
Plasma NO3- levels at visit 1 (V1) and the high and low NO3- beverage trials. Post NO_3_- levels at V1 were obtained two hours post consumption of a high NO_3_- beverage. Plasma NO_3_- levels at the high and low NO_3_- beverage trials were obtained two hours post consumption of the respective beverage. Plasma NO_3_- levels were not significantly different when comparing the high NO_3_- beverage trial values to the post consumption values at V1 (p = 1.0) or when comparing the low NO_3_- beverage trial values to the pre-consumption values at V1 (p = 1.0). Values are represented as median values with box ends representing the 25th and 75th percentiles and error bars representing the 5th and 95th percentiles. Dotted lines represent the mean values, and individual dots represent subject values.

**Fig 4:**
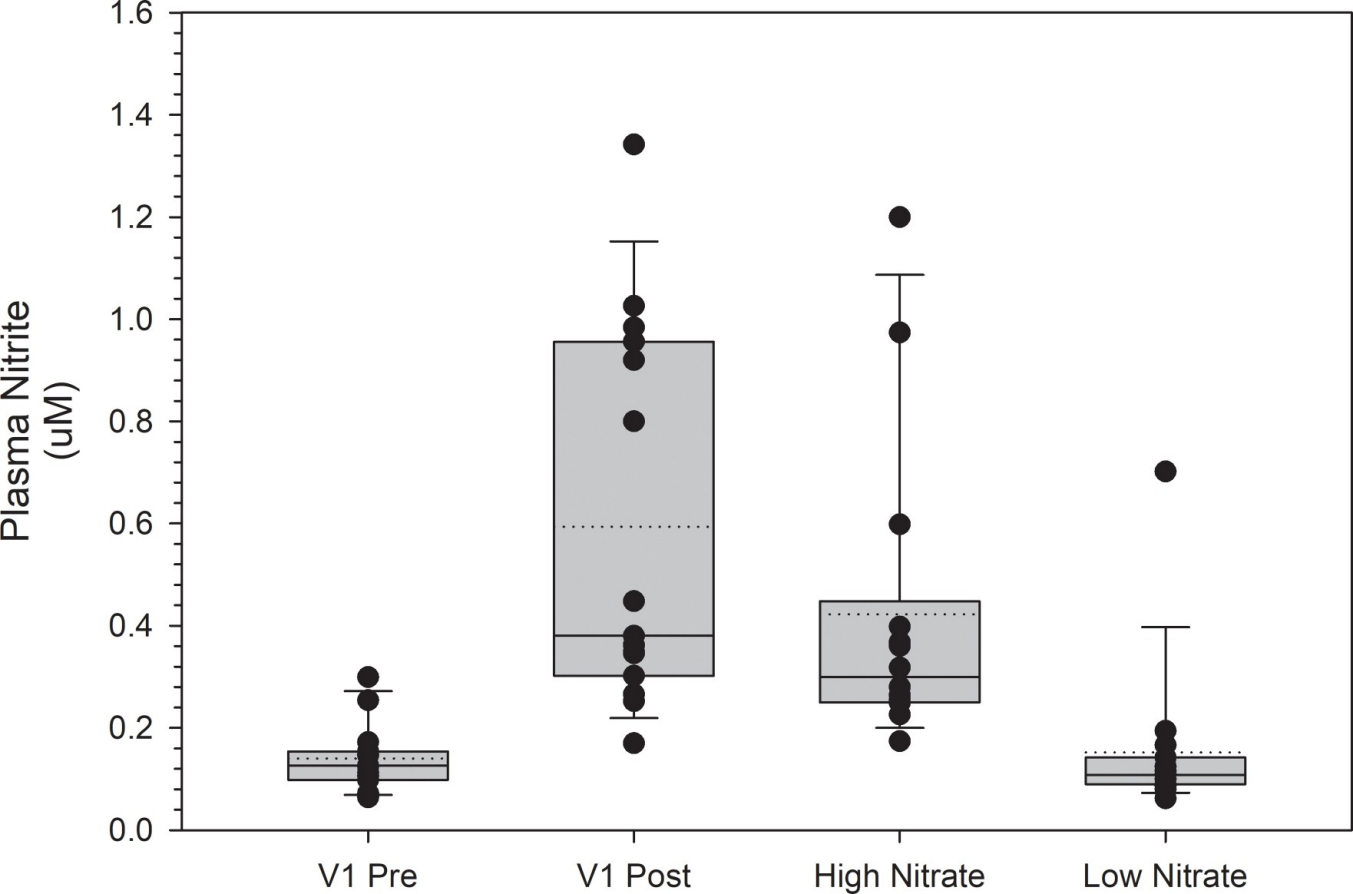
Plasma NO2- levels at visit 1 (V1) and the high and low NO3- beverage trials. Post NO_2_- levels at V1 were obtained two hours post consumption of a high NO_3_- beverage. Plasma NO_2_- levels at the high and low NO_3_- beverage trials were obtained two hours post consumption of the respective beverage. Plasma NO_2_- levels were not significantly different when comparing the high NO_3_- beverage trial values to the post consumption values at V1 (p = 1.0) or when comparing the low NO_3_- beverage trial values to the pre-consumption values at V1 (p = 1.0). Values are represented as median values with box ends representing the 25th and 75th percentiles and error bars representing the 5th and 95th percentiles. Dotted lines represent the mean values, and individual dots represent subject values.
