# Supplementary material for: A randomized controlled trial of nitrate supplementation in well-trained middle and older-aged adults
Source: PLoS One. 2020 Jun 23;15(6):e0235047. doi: 10.1371/journal.pone.0235047 (PMC7310701; doi:10.1371/journal.pone.0235047)
Supplement: S1 File — (PDF) [file pone.0235047.s002.pdf]

Date: Friday, January 17, 2020 2:28:15 PM

Print Close

View: RC SF_Protocol Textboxes

Protocol Textboxes

1.0 Background—describe the scientific background for this study as it relates to your application.
Nitric oxide has been identified as an important biological messenger involved in a number of physiological processes. It is produced from the amino acid L-arginine and molecular oxygen by nitric oxide synthases or the more recently identified nitrate→nitrite→nitric oxide pathway. Dietary nitrate has been shown to be a potential neutraceutic agent to improve exercise performance. Nitrate can be found in green leafy vegetables and is particularly abundant in beetroot. Dietary nitrate is reduced to bioactive nitrite by facultative bacteria found in the saliva and then to nitric oxide via additional pathways. The nitrate→nitrite→nitric oxide pathway has been shown to be involved in a number of physiological processes that could account for the improved exercise response following nitrate ingestion. The benefits of nitrate as an ergogenic aid have not been conclusively demonstrated, since numerous factors have been shown to influence its efficacy. These include age, training level, dosage and the mode, duration and intensity of the exercise.

Research with healthy younger moderately trained adults has repeatedly shown that ingestion of dietary nitrate can reduce the oxygen cost of submaximal exercise and improve exercise performance during high intensity exercise. Conversely, research with younger more highly trained endurance athletes is equivocal as some studies have failed to demonstrate an improvement in exercise performance following nitrate ingestion. There are several potential reasons for these conflicting results. First, well trained elite younger athletes may consume higher levels of dietary nitrate as a result of the increased daily energy intake related to their increased daily energy expenditure. These athletes are also thought to have increases in nitric oxide synthases, thus obviating the need for the nitrate→nitrite→nitric oxide pathway. Finally, elite endurance athletes exhibit a lower proportion of type II muscle fibers which are more responsive to nitrate supplementation.

The effects of nitrate have also been shown to be influenced by age. It has been documented that there are alterations in nitric oxide metabolism in older adults thought to result from impairment of the nitric oxide synthases pathway. We have shown that consumption of a supplement high in nitrate, such as beetroot juice, leads to elevated plasma nitrite levels and may help restore nitric oxide metabolism in older adults. As such, nitrate supplementation may improve physical function and exercise capacity in older adults. Our research, along with that of others has shown nitrate supplementation to have positive effects in older adults with chronic diseases. However, research examining the effects of dietary nitrate on exercise performance in healthy older adults is scarce. Presently, it is unclear as to whether nitrate supplementation is beneficial to exercise performance in older adults, and there is no data examining the effect of nitrate supplementation on exercise performance in active older adults. An examination of the National Health and Nutrition Examination Survey (https://www.cdc.gov/nchs/nhanes/index.htm) data shows that 36 percent of adults between the ages of 40 and 49, 28 percent of adults between 50 and 59 and 17 percent of adults between 60 and 69 reported that they engaged in strenuous activity at least once in the 30 days prior to being interviewed, and even greater percentages reported engaging in moderate activity. Therefore, it would be of interest to examine the effect of chronic nitrate supplementation of exercise performance in physically active higher functioning older adults.

2.0 Purpose—describe the purpose of your research. What question(s) are you trying to answer? Include your hypothesis(es) in this section, if applicable.
The primary aim of this investigation will be to evaluate the effect of short-term supplementation with a beetroot juice high in nitrate on exercise performance (as measured by submaximal constant work rate exercise capacity time) in healthy active adults aged 40 and over as compared to a beetroot juice low in nitrate.

We hypothesize that supplementation with a beetroot juice high in nitrate will improve the exercise capacity of physically active adults between the ages of 40 and 65.

3.0 Methods—

- **describe the setting where the research will occur**
- **describe what participants will be asked to do in this study**

- **include all steps participants will follow**
- **describe what constitutes the data and how it will advance your research question**

The proposed study will be a double blind, cross over study (see attached schematic). Subjects will complete one screening visit and four subsequent follow-up visits. During the screening visit (visit 1), subjects will read and sign the informed consent. They will then complete a contact, demographic and health questionnaire. They will also complete a physical activity questionnaire. A 3-4 ml venous blood sample will then be obtained to determine baseline plasma nitrate and nitrite levels. Subjects will then consume 4 ounces of Isagenix AMPED NOx which contains ~ 8 to 10 mmoles of nitrate. Approximately two hours later, a second 3-4 ml venous sample will be obtained for the determination of plasma nitrate and nitrite levels. Subjects that respond to the nitrate dosing by exhibiting a 100 percent increase in plasma nitrite will be asked to return for a second visit. In addition, subjects Our previous research has shown that approximately 20 percent of older adults consuming nitrates do not exhibit an increase in plasma nitrite levels; therefore, we will screen subjects to ensure that subjects respond to the nitrate ingestion and exhibit an increase in nitrite levels. We will also use the Global Physical Activity Questionnaire and score it to determine if the subjects engage in at least 150 minutes of moderate intensity physical activity or 75 minutes of vigorous physical activity per week. Subjects that do not meet these levels will be excluded from further participation. We have also added a question asking participants if they have competed in any competitive running or cycling events within the past two years. Those that respond no to both queries will also be excluded. All screening will be completed in person.

During visit 2, subjects will perform an incremental exercise test on an electronically braked cycle ergometer for the determination of their maximal work rate and oxygen consumption. During the test, the pedaling resistance will be increased every minute and expired gases will be collected via a mouthpiece for the determination of maximal oxygen consumption. The test will last about 10 to 15 minutes and will stop when subjects are no longer able to continue pedaling at prescribed work rate. The subject’s heart rate and rhythm will be monitored continuously via electrocardiography. Additionally, blood pressure and the subject’s level of effort will be measured before, during and after the test.

Approximately 3 to 4 days after the completion of the maximal exercise test, subjects will complete visit 3 which will consist of a practice submaximal constant work rate exercise test at 75 percent of the maximal work rate achieved during the graded exercise test at visit 2. The subjects will be asked to pedal for as long as they can. If they are still pedaling after 30 minutes, the resistance will be increased by 5 percent of the maximum work rate every 15 minutes until the subjects are no longer able to maintain the prescribed work rate. During this test, expired gases will be collected, heart rate, heart rhythm, blood pressure, the subject’s level of effort and level of tissue oxygenation will be measured.

Subjects who successfully complete the screening visit (visit 1), the maximal exercise test (visit 2) and the practice submaximal constant work rate exercise test (visit 3) will be randomized into the double-blind, cross-over treatment portion of the study. Randomization into the treatment sequences (AB and BA) will occur via a web based random number generator. A staff member not involved in data collection will arrange for the the packaging of study beverages and distribution to study participants. In this portion of the study, subjects will receive 1 of 2 treatments. Treatment 1 will consist of 7 days of beetroot juice high in nitrates followed by a submaximal constant work rate exercise test (visit 4) and then 7 days of a beetroot juice low in nitrates followed by a submaximal constant work rate exercise test (visit 5). Treatment 2 will consist of 7 days of a beetroot juice low in nitrates followed by a submaximal constant work rate exercise test (visit 4) and then 7 days of a beetroot juice high in nitrates followed by a submaximal constant work rate exercise test (visit 5). Administration of each treatment will be randomized and separated by a 7 day wash-out period. For each treatment, subjects will be asked to consume four ounces of the beverage daily at the same time of day. On day seven of each treatment, subjects will be asked to consume this beverage one and a half hours prior to their scheduled visit. Subjects will receive an email or text message daily from a staff member reminding them to drink their beetroot juice. On the seventh day of beetroot juice consumption, subjects will receive a call from the study staff reminding them of their visit and that they should consume the beetroot juice one and a half hours prior to the visit.

During visits 4 and 5, a 3-4 ml venous blood sample will be obtained to determine plasma nitrate and nitrite levels. Subjects will then complete a submaximal constant work rate exercise test at 75 percent of the maximal work rate achieved during the graded exercise test. The work rate will be set on the cycle ergometer and the subjects will be asked to pedal for as long as they can at that work rate. While unlikely, if they are still pedaling after 75% of their maximal work rate at 30 minutes, the resistance will be increased by five percent of the maximum work rate every 15 minutes until the subjects are no longer able to maintain the prescribed work rate. During each test, expired gases will be collected, heart rate, heart rhythm, blood pressure and the subject’s level of effort will be measured. Subjects will also be asked to rate the palatability of the beetroot juice beverage they consumed using a palatability questionnaire

Exercise time during the submaximal constant work rate tests during visits 4 and 5 will be the primary

outcome for this trial. In addition we will measure oxygen consumption, blood pressure, heart rate, perceived level of effort and tissue oxygenation during these tests.

AMPED NOx (Isagenix, LLC) will be used as the high nitrate beetroot juice beverage and V8 Purple Power (Campbell Soup Company) will be used as the low nitrate beetroot juice beverage. Dr. Dany Kim-Shapiro's lab has found that four ounces of AMPED NOx contains ~ 8 to 10 mmoles (580 to 680 mg) of nitrate, and four ounces of V8 Purple Power contains ~ 3 mmoles (190 mg) of nitrate.

Any individuals who may have acute illnesses during the testing time will be rescheduled for their testing. Should they elect not to continue, they would not be eligible for compensation.

A. Do your methods involve deception (intentionally interacting, communicating, or intervening with study participants in a way that produces false beliefs)?

○ Yes ● **No**

B. Do your methods involve any of the following?

☐ Biological material (e.g., blood, saliva, tissue, urine) will be collected and/or stored.

☑ Drug, chemical, metabolite, nutritional substance, biological agent, nutraceutical, or other substance (whether regulated/approved by the FDA or not) will be administered.

☐ Medical device, instrument, machine, computer program or other device (whether regulated/approved by the FDA or not) will be used.

☐ Placebo procedure or treatment will be used as a control.

C. Does this study meet the definition of a Clinical Trial?

○ Yes ○ No

4.0 Measures- click the Add button to upload instruments, surveys, interview questions, observation checklists, etc. Upload the most current clean copy of each document only (redlined versions should be uploaded in item 5.0 below).

Name	Modified	Version
BRJ Palatability Survey_Clean_Ver1.docx	11/21/2017 4:23 PM	0.01
Contact, Demo and Health Questionnaire_clean_V1.doc	11/21/2017 4:23 PM	0.01
Global Physical Activity Questionnaire.pdf	10/12/2017 4:39 PM	0.01
Hypothyroid Approval Letter.docx	4/11/2018 4:43 PM	0.01

5.0 Redlined Measures- If you have been asked to make changes to any measure(s), click the Add button to upload the redline version(s) here.

Name	Modified	Version
BRJ Palatability Survey_Redline_Ver1docx.docx	11/21/2017 4:22 PM	0.01
Contact, Demo and Health Questionnaire_redline_V1.doc	11/21/2017 4:22 PM	0.01

6.0 Optional Support Documents - click the Add button to upload any documents you are submitting in support of this application (e.g., References, Charts, Diagrams, etc.).

Name	Modified	Version
Blood Borne Pathogen Training Certification.pdf	10/30/2017 3:23 PM	0.01
Isagenix Trial Schematic.docx	10/9/2017 4:00 PM	0.01
